# Complex network model indicates a positive effect of inspiratory muscles pre-activation on performance parameters in a judo match

**DOI:** 10.1038/s41598-021-90394-1

**Published:** 2021-05-27

**Authors:** Carolina Cirino, Claudio A. Gobatto, Allan S. Pinto, Ricardo S. Torres, Charlini S. Hartz, Paulo H. S. M. Azevedo, Marlene A. Moreno, Fúlvia B. Manchado-Gobatto

**Affiliations:** 1 Laboratory of Applied Sport Physiology, School of Applied Sciences, University of Campinas, 1300 Pedro Zaccaria St, Limeira, Sao Paulo 13484-350 Brazil; 2 Department of ICT and Natural Sciences, Norwegian University of Science and Technology, Ålesund, Norway; 3 Postgraduate Program in Human Movement Sciences, Methodist University of Piracicaba, Piracicaba, Sao Paulo Brazil; 4 Department of Human Movement Sciences, Federal University of São Paulo, São Paulo, Brazil

**Keywords:** Mathematics and computing, Physiology, Respiration

## Abstract

This study investigated the effects of inspiratory muscle pre-activation (IM_PA_) on the interactions among the technical-tactical, physical, physiological, and psychophysiological parameters in a simulated judo match, based on the centrality metrics by complex network model. Ten male athletes performed 4 experimental sessions. Firstly, anthropometric measurements, maximal inspiratory pressure (MIP) and global strenght of the inspiratory muscles were determined. In the following days, all athletes performed four-minute video-recorded judo matches, under three conditions: without IM_PA_ (CON), after IM_PA_ at 15% (IM_PA_15), and at 40% (IM_PA_40) of MIP using an exerciser device. Blood lactate, heart rate and rating of perceived exertion were monitored, and the technical-tactical parameters during the match were related to offensive actions and the time-motion. Based on the complex network, graphs were constructed for each scenario (CON, IM_PA_15, and IM_PA_40) to investigate the Degree and Pagerank centrality metrics. IM_PA_40 increased the connectivity of the physical and technical-tactical parameters in complex network and highlighted the combat frequency and average combat time in top-five ranked nodes. IM_PA_15 also favoured the interactions among the psychophysiological, physical, and physiological parameters. Our results suggest the positive effects of the IM_PA_, indicating this strategy to prepare the organism (IM_PA_15) and to improve performance (IM_PA_40) in judo match.

## Introduction

Inspiratory training has been shown to improve sports performance^1–4^.The muscle pre-activation, which promotes post-activation potentiation, corresponds to a previously performed muscle activity that contributes to potentiate performance in the main activity^5,6^. In the same way, the inspiratory muscles warm-up, here called inspiratory muscles pre-activation (IM_PA_), showed positive effects to improve maximal inspiratory pressure (MIP)^7–13^ and sports performance in rowing^8^, badminton^9^, intermittent running^11^, swimming^14^, anaerobic test^15^, and long-distance running^16^. However, some investigations have not identified the same effect for ventilatory and metabolic responses^8,9,12,14,16–19^, except for tissue saturation index of active muscles^18^, respiratory rate^20^, and perception of dyspnea^16^. Generally, IM_PA_ is composed by inspiratory efforts performed on a respiratory exerciser device with an intensity determined by the percentage of the individual's MIP^7–16^.

Modalities such as judo, with higher request on the upper limbs present high and double mechanical demand on inspiratory muscles^21^ and can favour the induction of fatigue in these muscles^22–24^, activating the metaboreflex^25^.The IM_PA_ can be a resource to optimize performance during the judo match since the constants imbalances of offensive and defensive actions in different positions and directions^26,27^ can activate inspiratory muscles for ventilation mechanics and trunk stabilization^28,29^. The use of this strategy can potentiate the action of the inspiratory muscles in the control of the trunk, improving the quality of the movements of the applied techniques and influencing the result of the score during the combat^30^. Merola et al.^31^ applied the high-intensity IM_PA_ before a specific test for judo with projection techniques (*Special Judo Fitness Test*). Even though that study has not found positive results on test performance, the evidence suggests that inspiratory efforts applied using lower intensities before the exercise may demonstrate the benefits of this strategy.

In addition, the cause-effect analyses of traditional statistics seem insufficient to understand multifactorial responses to performance^32^, including in combat sports. The complex network can investigate the relationships between the performance parameters of athletes in different modalities^33–35^. Sports science lacks studies on the interaction among technical-tactical parameters and physical and physiological aspects under the intervention of strategies to improve performance in combat sports. Therefore, this study aimed to investigate the effects of inspiratory muscle pre-activation on the interactions among technical-tactical, physical, physiological, and psychophysiological parameters in a simulated judo matches, based on the analysis of centrality measures for complex network models. Considering previous studies involving acute inspiratory load strategies and the judo characteristics, we hypothesize that IM_PA_ at 40% of MIP (IM_PA_40) will be promote positive effects on the technical-tactical, physiological, and psychophysiological parameters of a simulated judo match. Additionally, the centrality measures obtained by complex network models will be able to identify the impact of the IM_PA_ on judo match, improving the interaction and connectivity among the technical-tactical, physical, physiological, and psychophysiological parameters, especially after the IM_PA_40.

## Methods

### Subjects

Ten male judo athletes, medallists in official competitions (2018–2019) at state and national levels participated in the study. The athletes’ profile is described in Table 1 (see “Results” section). The athletes mentioned no metabolic, cardiovascular, respiratory, or orthopaedic disease and no use of medications or drugs. All athletes were evaluated in the pre-competitive period and aware of sleep, food, and physical training conditions before each test.

**Table 1:** Athletes’ profile, anthropometric characteristics and body composition and inspiratory measure of the participants.

Athletes’ profile	
Age (years)	22 ± 1
Judo practice time (years)	15 ± 2
Graduation in judo	8 black belts, 2 brown belts
**Anthropometric characteristics and body composition**	
Height (cm)	176.2 ± 2.0
Wingspan (cm)	179.7 ± 2.5
Body mass (kg)	77.8 ± 3.7
Fat mass** (%)	10.7 ± 1.1
Transverse thoracic diameter (mm)	30.2 ± 0.7
Anteroposterior thoracic diameter (mm)	21.6 ± 0.4
Neck circumference (cm)	38.7 ± 0.6
Arm circumference flexed (cm)	35.8 ± 0.9
Thorax circumference (cm)	95.8 ± 1.9
Waist circumference (cm)	80.7 ± 2.3
Abdominal circumference (cm)	82.3 ± 2.2
Hip circumference (cm)	98.8 ± 2.0
Thigh circumference (cm)	55.7 ± 1.4
**Inspiratory measures**	
MIP (cmH_2_O)	157.0 ± 6.1
Peak values S-index (cmH_2_O)	121.8 ± 5.0
Average values S-index (cmH_2_O)	106.9 ± 8.1
Peak values PIF (L/s)	6.8 ± 0.3
Average values PIF (L/s)	6.0 ± 0.4
Peak values volume (L)	3.4 ± 0.2
Average values volume (L)	2.9 ± 0.3

**7 Skinfolds^36^. Results are expressed as mean values ± SEM. *MIP* muscle inspiratory pressure, *PIF* peak inspiratory flow.

### Experimental design

**Figure 1:**
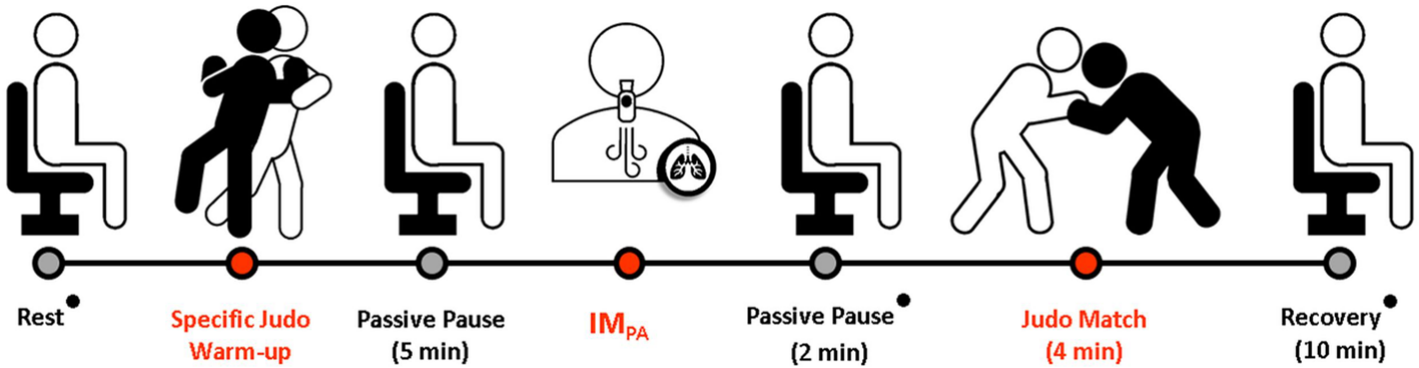
Timeline for the execution of the assessment sessions, considering the Inspiratory Muscles Pre-Activation (IM_PA_) before a judo match and passive recovery. (filled circle) Physiological parameters collected at rest, pre and post combat, and recovery at each 2 min until 10 min.

Four assessment sessions were performed, separated by 24–48 h. In the first section, all subjects (n = 10) received information about the experimental design and signed the consent form. In addition, they performed the assessment of anthropometric and inspiratory measures, and familiarization with the equipment and protocols. The other sessions were performed, in random order, to evaluate the effects of inspiratory muscles pre-activation (IM_PA_) previously to a judo match (Fig. 1). All subjects performed these sessions in three different conditions: (1) control protocol, in which the judo match was carried out without the IM_PA_ (CON); (2) judo match preceded by IM_PA_ using 15% of MIP (IM_PA_15); (3) after IM_PA_ using 40% of MIP (IM_PA_40). The participants performed a specific judo warm-up composed of mobility exercises for all joints (20 repetitions) and 2 sets of 20 techniques without projection (*Uchikomi*) on each side with 30 s of interval before each intervention, followed by 5 min of passive pause and application of the IM_PA_ protocol. After 2 min of passive pause, the athletes performed a judo match, followed by 10 min of recovery. In control protocol, the athletes completed only the specific judo warm-up and started the match immediately after the passive pause of 5 min. Information on the inspiratory loads and the possible effects of the protocols was omitted from the participants.

### Inspiratory measures

The maximal inspiratory pressure (MIP) was obtained through the inspiratory effort initiated from the residual volume after maximum expiration^37^. Participants remained seated and performed at least 5 maximum inspiration efforts with a 1-min interval^38^, three acceptable and two reproducible (that is, a variation of values ≤ 10%). The efforts were sustained by at least 1 s to register the highest inspiratory pressure, considering the measure of greatest value. The analog manovacuometer (± 300 cmH_2_O) (Ger-ar®, Brazil) was used for these measurements. After 30 min, the global strength of the inspiratory muscles (S-index) of the participant in the standing position was evaluated. The participants performed a sequence of 30 inspirations in an inspiratory muscle exerciser (POWERbreathe® K5 model, IMT Technologies Ltd., Birmingham, UK), with verbal encouragement to inspire greater air capacity^39^. The average and maximum values of the global force (S-index), peak inspiratory flow (PIF) and volume measures were obtained using the Breathe-Link Version 1.1 software. All participants used a nose clip in both tests.

### Inspiratory muscle pre-activation (IM_PA_)

According to previous studies that applied warm-up to inspiratory muscles^7,9,13,14^, our protocols were conducted under three conditions. The control protocol was characterized by the judo match without IM_PA_. On the other hand, in IM_PA_15 and IM_PA_40 sessions, the athletes were submitted to these acute strategies using loads equivalent to 15% and 40% of the MIP, respectively. The IM_PA_ interventions were performed with an inspiratory muscle exerciser POWERbreathe® K5 model (IMT Technologies Ltd., Birmingham, UK). During the procedure, the subjects remained in a standing position and performed 2 sets of 15 maximum inspirations (1-min interval between sets), maintaining the diaphragmatic inspiratory muscle pattern. All repetitions were monitored by the equipment's software.

### Technical-tactical parameters

The simulated judo matches followed the official rules (2018–2020)^30^. To guarantee the same conditions in all assessment sessions, the matches lasted 4 min, independent of the score achieved. The participants performed all matches with the same opponent of equivalent technical level (difference in body mass < 10%)^40^. The technical-tactical parameters were obtained by notational analysis from video-recorded. Offensive actions: number of attacks, number of scores (*Ippon and Wazari*) and penalties (*Shido*), for effectiveness ((number of scores/number of attacks) × 100)^41^ and efficiency ((number of *Ippon* × 10) + (number of *Wazari* × 7) for 1 combat^42^. Time-motion: combat frequency, average combat time, average and total values of pause time, standing combat time and groundwork combat time; time between attacks and effort-pause ratio (ratio between the average combat time and the average pause time)^27,43^.

### Physiological and psychophysiological parameters

The physiological parameters of blood lactate [Lac] and heart rate (HR) were monitored at rest, pre- and post-combat, and at each 2 min unitl 10 min of recovery. Heart rate (bpm) was continuously recorded every second by a monitor (Polar® model V800, Finland). Blood samples (25 µL) were extracted from the ear lobe with a heparinized capillary and deposited in microtubes (Eppendorf, 1.5 ml) containing 50µL of 1% sodium fluoride and frozen at – 20 °C before reading lactate concentrations. The blood lactate concentrations were determined on a lactate analyser (YSI-2300-STAT-Plus™, Yellow Springs, OH, USA). The Rating of Perceived Exertion (RPE)^44^ was applied as a psychophysiological parameter.

### Complex networks analysis

The analyses of the interactions between the performance parameters of a judo match under IM_PA_ intervention were obtained from a complex network model, in each scenario (CON, IM_PA_15, and IM_PA_40). Each complex network was composed of set of undirected weighted graph G = (V, E, w), where the 52 vertices V (nodes) correspond to the technical-tactical, physiological, and psychophysiological parameters of the judo match and the common parameters to the three scenarios that consist of the characteristics anthropometric and body composition, sport profile and the inspiratory measures of athletes. The edges (E) represent the interactions mediated by the values of "r" statistically significant Spearman correlations (*p* ≤ 0.05); and w is the weight function^45^. The topology of the networks can be seen in Fig. 4. The centrality metrics of Degree and Pagerank were applied to the scenarios^34,35,46^. The degree metric representing the number of edges of the node that connects to the other nodes. The influence of one node on the others in the network is highlighted by the Pagerank metric^35^. Data processing for the elaboration of the complex network was performed using a specific algorithm in MATLAB environment. The analyses were obtained by software Gephi (0.9.2 version) implemented in the JAVA programming language applying the Fruchterman-Reingold layout^47^ to construct the graphs.

### Statistical analysis

The results are described as mean and standard error of the mean (SEM). Non-parametric statistics were adopted, since the normality of the data was not attested by the Shapiro–Wilk test. Homogeneity was verified by the Levene test. The Friedman test followed by the Bonferroni post-hoc test, were applied to compare the technical-tactical, physiological, and psychophysiological parameters in each intervention performed by all athletes (CON × IM_PA_15 × IM_PA_40). Comparisons of physiological and psychophysiological responses (before and after the judo match) were performed using the Friedman test for repeated measures, observing the effect of IM_PA_ and time. Spearman's correlation was used between all performance parameters for the elaboration of complex network models. The level of significance adopted was *p* ≤ 0.05.

### Ethics approval

This study was conducted in agreement within the ethical recommendations of the Declaration of Helsinki, and all experiments were approved by the Research Ethics Committee of The School of Medical Sciences (protocol number 16561019.2.0000.5404). Participants were only evaluated after having received information about the experimental procedures and risks and signing an informed consent form.

## Results

Table 1 shows the athletes’ profile, anthropometric characteristics, body composition, and inspiratory measurements (mean values ± SEM).

### Traditional analysis of technical-tactical parameters

**Figure 2:**
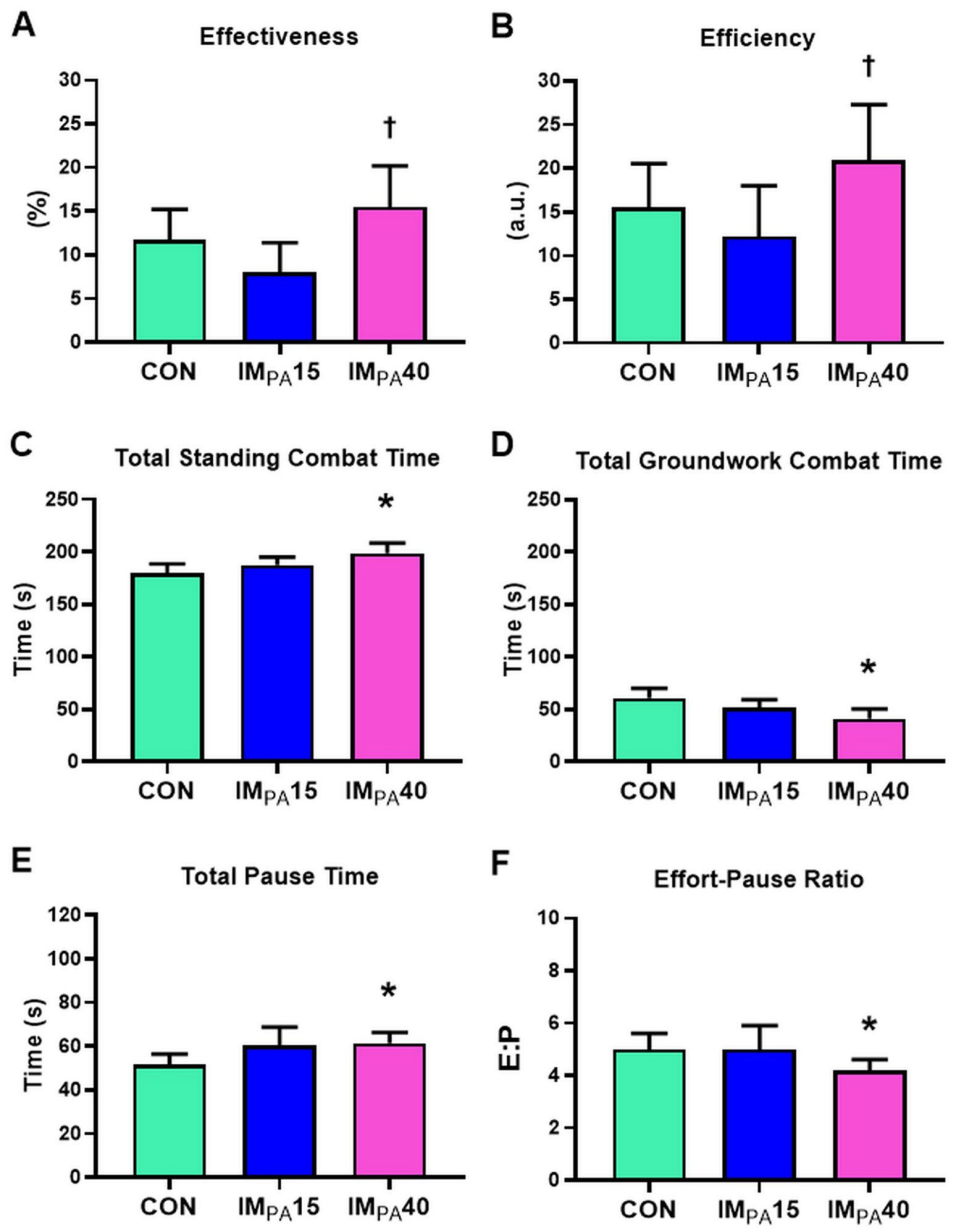
Technical-tactical parameters in the judo match. Results are presented as mean ± SEM. (**A**) Effectiveness (%); (**B**) Efficiency (a.u.); (**C**) Total Standing Combat Time (s); (**D**) Total Groundwork Combat Time (s); (**E**) Total Pause Time (s); (**F**) Effort-Pause Ratio (E:P). † Significant difference (*p* ≤ 0.05) in comparison to IM_PA_15. *Significant difference (*p* ≤ 0.05) in comparison to CON.

The number of attacks, *Wazari* Scores, and penalties for *Shido* did not differ among the protocols (Table 2). However, IM_PA_40 significantly increased *Ippon* Scores compared to IM_PA_15. Figure 2A,B shows that effectiveness (15.5 ± 4.7%) and efficiency (20.9 ± 6.4) were also higher by IM_PA_40 when compared to IM_PA_15 (8.0 ± 3.4%; *p* = 0.020 and 12.2 ± 5.8; *p* = 0.020, respectively). In time-motion, the parameters related to combat periods (average values) were not changed by the interventions (Table 2). IM_PA_40 provided changes in relation to the total values of standing combat (198.7 ± 9.7 s), groundwork combat (41.4 ± 9.0 s) and pause (61.4 ± 4.9 s) compared to CON (179.6 ± 9.0 s; *p* = 0.022, 60.8 ± 9.4 s; *p* = 0.005 and 51.8 ± 4.6 s; *p* = 0.042, respectively) (Fig. 2C–E). The effort-pause ratio (Fig. 2F) showed a ratio of 3.6: 1 in IM_PA_40, while a ratio of 4.3:1 (*p* = 0.028) was observed in control. We emphasize that there were no significant differences between CON and IM_PA_15 for any of the technical-tactical parameters using the traditional statistical analysis.

**Table 2:** Technical-tactical parameters of offensive actions of combat and time-motion and physiological parameters of blood lactate [Lac], heart rate (HR) and psychophysiological parameters of rating of perceived exertion (RPE), described in mean ± SEM.

	CON	IM_PA_15	IM_PA_40	*p* value
**Technical-tactical parameters**				
Offensive actions				
Attacks (a.u.)	16 ± 2	15 ± 2	16 ± 2	0.836
* Ippon* score (a.u.)	1 ± 0	1 ± 0	2 ± 1^a^	0.010
* Wazari* score (a.u.)	1 ± 0	1 ± 0	1 ± 0	1.000
* Shido* penalty (a.u.)	0 ± 0	0 ± 0	0 ± 0	0.584
Time-motion				
Combat frequency (a.u.)	8 ± 1	9 ± 1	8 ± 1	0.618
Average combat time (s)	31.6 ± 2.4	32.5 ± 4.3	30.1 ± 2.5	0.798
Average pause time (s)	7.5 ± 0.4	8.1 ± 0.5	8.4 ± 0.4	0.223
Average standing combat time (s)	24.0 ± 2.7	25.8 ± 3.8	25.3 ± 3.1	0.741
Average groundwork combat time (s)	12.3 ± 1.5	13.7 ± 2.1	8.5 ± 1.8	0.067
Time between attacks (s)	17.6 ± 2.6	18.6 ± 2.1	16.1 ± 1.6	0.836
**Physiological parameters**				
Blood lactate				
Rest [Lac] (mM)	1.1 ± 0.1	1.0 ± 0.1	1.1 ± 0.1	0.497
Pre-combat [Lac] (mM)	2.0 ± 0.3	1.5 ± 0.2	1.7 ± 0.3	0.273
Post-combat [Lac] (mM)	9.5 ± 1.0	9.4 ± 1.0	9.3 ± 0.8	0.670
∆ [Lac] (mM)	7.5 ± 1.0	7.9 ± 0.9	7.6 ± 0.8	0.905
Peak [Lac] (mM)	10.1 ± 1.0	9.9 ± 1.0	9.8 ± 0.9	0.497
Time to reach the peak [Lac] (min)	2.2 ± 0.6	2.2 ± 0.6	1.8 ± 0.7	0.792
Rate of decay [Lac] (%)	23.6 ± 3.9	28.8 ± 3.1	25.7 ± 4.0	0.273
Heart rate				
Rest HR (bpm)	75 ± 5	76 ± 3	73 ± 4	0.614
Pre-combat HR (bpm)	119 ± 5	107 ± 6	107 ± 7	0.062
Post-combat HR (bpm)	181 ± 3	178 ± 4	179 ± 2	0.482
∆ HR (bpm)	62 ± 4	71 ± 6	72 ± 6	0.223
Rate of decay HR (%)	44.5 ± 1.5	43.5 ± 1.5	45.9 ± 1.5	0.584
** Psychophysiological parameters**				
Rating of perceived exertion				
Rest RPE (a.u.)	9 ± 1	9 ± 1	9 ± 1	0.325
Pre-combat RPE (a.u.)	9 ± 1	9 ± 1	9 ± 1	0.891
Post-combat RPE (a.u.)	16 ± 0	16 ± 0	16 ± 0	0.875
∆ RPE (a.u.)	7 ± 1	7 ± 1	7 ± 1	0.969
Rate of decay RPE (%)	38. 2 ± 3.6	36.7 ± 3.7	38.1 ± 4.0	0.666

^a^Significant difference (*p* ≤ 0.05) in comparison to IM_PA_15.

∆ = (values post-combat − values pre-combat).

Rate of decay (recovery) = ((maximum − minimum/maximum) × 100).

### Traditional analysis of physiological and psychophysiological parameters

**Figure 3:**
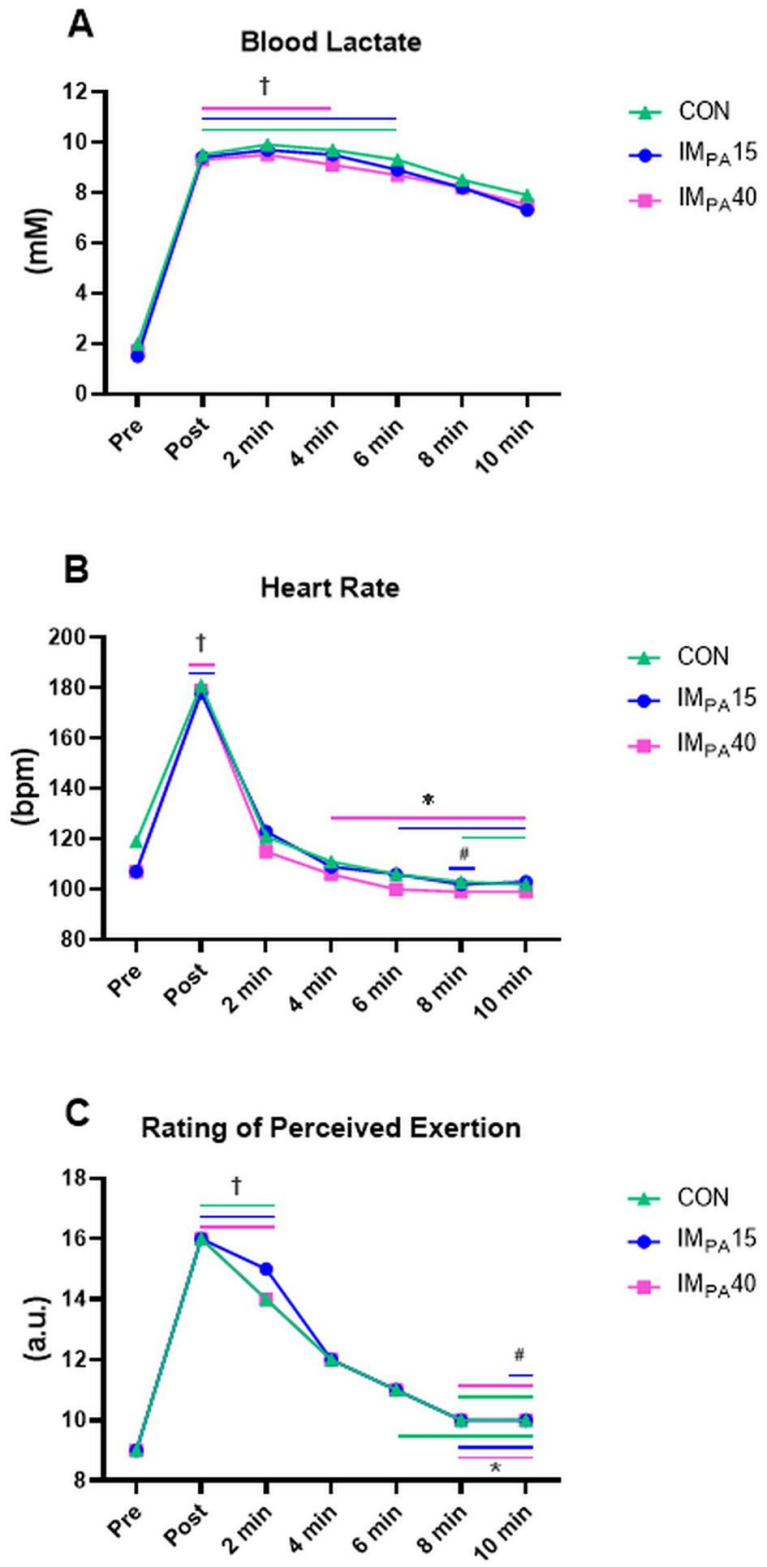
Physiological and psychophysiological parameters pre, post and recovery a judo match. The results are presented in mean values. (**A**) Blood lactate (mM); (**B**) Heart rate (bpm); (**C**) Rating of perceived exertion (a.u.). † Significant difference compared to the pre-combat moment; *significant difference compared to the post-combat moment; # significant difference compared to the 2^nd^ min post-combat (*p* ≤ 0.05).

The physiological and psychophysiological parameters were not affected by IM_PA_ (Table 2 and Fig. 3). Figure 3 demonstrated that [Lac], HR, and RPE were significantly different over the time of post-combat recovery in all interventions. The [Lac] was higher from the post-combat moment until the 6th min of recovery in the CON and until the 4th min in the IM_PA_15 and IM_PA_40 compared to the pre-combat moment (Fig. 3A). The HR (Fig. 3B) showed significantly higher values in the post-combat moment in relation to the pre-combat values in IM_PA_15 and IM_PA_40. The values of HR were significantly lower from the 4th min on IM_PA_40, from the 6th min on IM_PA_15 and only after the 8th min on the CON until the end of recovery. The RPE values after combat and in the 2nd min of recovery were significantly higher in the three protocols in relation to the pre-combat moment. In comparison with the post-combat moment, the RPE decreased significantly from the 6th (CON) and 8th (IM_PA_15 and IM_PA_40) until the 10th min. The 8th and 10th minute of CON and IM_PA_40 and 10th min of IM_PA_15, presented significantly lower values of RPE when compared to the 2nd minute recovery.

### Complex network analysis

**Figure 4:**
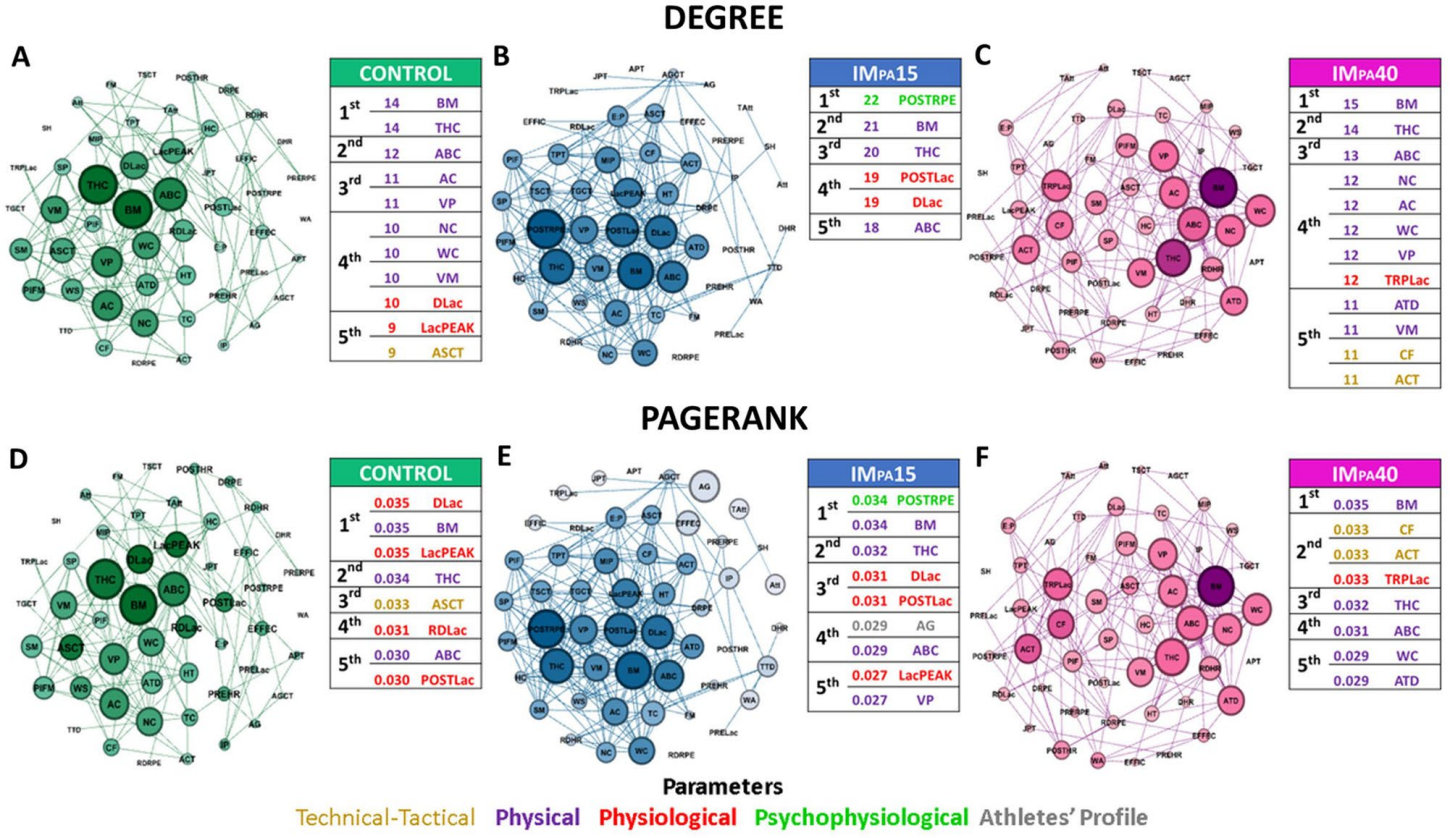
Three scenarios of a simulated judo match represented by graphs, considering the Degree and Pagerank centrality measurements: (**A**,**D**) Control; (**B**,**E**) IM_PA_15 e (**C**,**F**) IM_PA_40). The tables demonstrate top-five node results involving athletes' profile (grey), technical-tactical (yellow), physical (purple), physiological (red), and psychophysiological (green). AG: Age; JPT: Judo Practice Time; HT: Height; WS: Wingspan; BM: Body Mass; TTD: Transverse Thoracic Diameter; ATD: Anteroposterior Thoracic Diameter; NC: Neck Circumference; AC: Arm Circumference Flexed; TC: Thorax Circumference; WC: Waist Circumference; ABC: Abdominal Circumference; HC: Hip Circumference; THC: Thigh Circumference; FM: Fat Mass; MIP: Maximal Inspiratory Pressure; SM: S-index Mean; SP: S-index Peak; PIFM: Peak Inspiratory Flow (mean); PIF: Peak Inspiratory Flow (peak); VM: Volume (mean); VP: Volume (peak); PRELac: Pre-Combat Blood Lactate; POSTLac: Post-Combat Blood Lactate; DLac: Delta of Blood Lactate; LacPEAK: Peak of Blood Lactate; TRPLac: Time to Reach the Peak of Blood Lactate; RDLac: Rate of Decay of Blood Lactate; PREHR: Pre-Combat Heart Rate; POSTHR: Post-Combat Heart Rate; DHR: Delta of Heart Rate; RDHR: Rate of Decay of Heart Rate; PRERPE: Pre-Combat—Rating of Perceived Exertion; POSTRPE: Post-Combat—Rating of Perceived Exertion; DRPE: Delta of Rating of Perceived Exertion; RDRPE: Rate of Decay of Rating of Perceived Exertion; CF: Combat Frequency; ACT: Average Combat Time; TPT: Total Pause Time; APT: Average Pause Time; TSCT: Total Standing Combat Time; TGCT: Total Groundwork Combat Time; ASCT: Average Standing Combat Time; AGCT: Average Groundwork Combat Time; TAtt: Time between Attacks; E:P: Effort-pause ratio; Att: Attacks; EFFEC: Effectiveness; EFFIC: Efficiency; IP: *Ippon*; WA: *Wazari*; SH: *Shido.*

Figure 4 presented the Degree and Pagerank centrality measures, highlighting top-five results in each scenario (10% of 52 parameters), considering the equality between the parameters that presented metrics with the same value. CON presented 145 connections, IM_PA_15 showed 229, and IM_PA_40 returned 167 connections. Degree ranking showed that CON (Panel A) and IM_PA_40 (Panel C) were the scenarios with the highest participation among different performance parameters (11 and 12 nodes, respectively), with greater emphasis on physical parameters. IM_PA_15 (Panel B) indicated the participation of only 6 parameters but demonstrated a greater number of connections for the same node, highlighting postRPE (1st), followed by BM (2nd) and THC (3rd) with values above 20 connections. CON and IM_PA_15 presented 14 and 15 connections for the parameter ranked in 1st (BM). IM_PA_40 showing the physical parameters, followed by the technical-tactical and physiological parameters. IM_PA_15 classified the psychophysiological, physical, and physiological parameters. In Pagerank ranking, CON (Panel D) stressed the importance of the physiological parameters DLac and LacPEAK (1st). IM_PA_15 (Panel E) indicated postRPE and BM in 1st, emphasizing the physical and physiological parameters. IM_PA_40 (Panel F) presented BM as the main parameter followed by CF, ACT and TRPLac (2nd). Differently others, in this scenario important technical-tactical parameters were highlighted. For both metrics used, the physical parameters presented the greatest number of interactions and importance among all the performance parameters of a judo match, with emphasis on BM that was among the first two positions in the rankings in all scenarios.

## Discussion

To the best of our knowledge, this is the first study that analysed the effects of IM_PA_ on the interactions among the technical-tactical, physical, physiological, and psychophysiological parameters in a simulated judo match. Partially confirming our hypothesis, the IM_PA_40 improved the performance of *Ippon Scores*, effectiveness and efficiency compared with IM_PA_15, besides promoting changes in time-motion. However, this strategy did not modify the physiological parameters before and after the judo match, at least using traditional analysis. In an innovative way to judo modality, our study reinforces the complex network model to improve the data interpretations into athlete´s performance. Using this model, IM_PA_40 scenario showed interactions mainly among the physical and technical-tactical parameters important to judo match. Differently of the IM_PA_40, the acute load using 15% of the MIP did not highlight the technical-tactical parameters as top-five nodes in centrality metrics obtained by complex network. Despite that, IM_PA_15 showed the highest connectivity among physical and physiological parameters, suggesting that lower inspiratory load already prepare the organism of this athletes.

The quality of movements of techniques in standing and groundwork combat can influence the result of the score in a judo match^30^. Using the non-parametric statistical analysis, we observed the effect of IM_PA_40 on *Ippon* Scores (Table 2) and effectiveness and efficiency (Fig. 2A,B) when compared to IM_PA_15. In relation to CON, IM_PA_40 provided changes in the total values of standing combat, groundwork combat and pause. Although the official rules of judo advantage standing combat, especially when there is no objectivity of offensive actions on the groundwork^30^, changes in total standing and groundwork combat times (Fig. 2) are associated with previous results, since the positive effects on the offensive actions may have induced the choice of strategy to conduct the combat standing, in which the frequency of scores^48^ and efficiency^42^ are generally larger than in groundwork combat. The other time-motion parameters (Table 2) that did not differ among the protocols, indicate that there was no effect of IM_PA_15 or IM_PA_40 on the dynamics of the match. These results reflect the behaviour presented by the physiological and psychophysiological parameters (Fig. 3).

The [Lac], HR and RPE values (Fig. 3A–C) decreased during post-combat recovery but were not affected by IM_PA_ strategies. In line with our results, previous studies that applied the traditional IM_PA_ protocol in high-intensity efforts or in exhaustive tests did not observe differences in ventilatory and metabolic responses^8,9,12,14,17–19^^.^ High-intensity efforts cause fatigue in the inspiratory muscles, activating the metaboreflex^25^. The accumulation of metabolites caused by intense exercise stimulates the types III and IV afferent fibers of the inspiratory muscles, especially the diaphragm^49^. This process increases the sympathetic activity of the muscle that promotes adrenergic vasoconstriction, redistributing the blood flow from the active musculature to the respiratory muscles^50^. Here, at least through investigations using traditional analysis, the physiological and psychophysiological parameters were not altered regardless of the acute loads adopted (15 or 40% of MIP) suggesting that IM_PA_ was not able to significantly inhibit the metaborreflex, probably due to the ventilatory and postural demands of the inspiratory muscles during the judo match. On the other hand, these results reinforce that the positive effect of IM_PA_40 on the technical-tactical parameters was not explained exclusively by blood lactate, HR or RPE responses.

The literature indicates the inspiratory acute load at 15% of MIP as a placebo condition^8,9,14,18^. If considered this point, we observed the impact of the IM_PA_40 in judo match by traditional statistical analysis, without modifying physiological responses. In this way, probably the technical-tactical alterations could be associated with neural control of inspiratory muscles that assist in ventilatory mechanics and postural function^51^. The neural control of the inspiratory muscles can be activated automatically by the bulbospinal pathways and voluntarily, by corticospinal pathways^51^. Although without direct evidence, we could suggest that the resistance caused by IM_PA_40 during voluntary inspiration contribute to postural adjustment and the application of techniques during combat, since the diaphragm is also activated in tasks with upper limb movements^28^ and intercostal muscles in trunk rotations^29^. In line with this reasoning, studies have attributed the increase in MIP after IM_PA_ to improved intra and intermuscular coordination of inspiratory muscles^7,9–13^ considering that resisted inspiration can cause changes in the heavy chain of myosin that modulates the recruitment of motor units^52^. On the other hand, as follow discussed, the integrative analysis applied in our study did not confirm the IM_PA_15 as a placebo conditioning to judo match.

It is important emphasize that many studies involving acute muscle inspiratory protocols used 2 sets of 30 maximum inspirations with a load equivalent to 40% of MIP^7–11,14,18^. Here, we adopted IM_PA_ protocols as 2 sets of 15 maximum inspirations using the exercise device. Our choice was based on Merola et al.^31^ who investigated the Special Judo Fitness Test^53^ parameters under the intervention of an IM_PA_ (2 sets of 15 maximum inspirations, at 60% of MIP) and due our athletes having no previous experience with respiratory training. Accordingto our results, this method using fewer number of inspiratory repetitions, already provide benefits to judo athletes, which was confirmed by an integrative analysis as discussed below.

Complex networks (Fig. 4) demonstrated the effects of IM_PA_ in a simulated judo match, mainly on physical and technical-tactical parameters. IM_PA_15 and IM_PA_40 promoted greater connectivity among performance nodes (an increase of connections around 59.7% and 15.2%, respectively). The centrality metrics emphasized on the physical parameter’s BM and THC (see top-five nodes—Fig. 4). In a similar way, Gobatto et al.^35^ investigated two scenarios of laboratory and field tests in basketball players, highlighted the body mass and the vertical jump power (measure related to the lower limbs) by complex network analysis. The BM and THC are so important for judo, as athletes compete in matches divided by body mass and high levels of lower limb strength are required during the application of projection techniques^54^. Thus, THC can be an indirect method for measuring the muscle^55^, where the cross-sectional area of ​​the muscle is related to the ability to generate strength^56^.

The centrality metrics showed that IM_PA_15 presented more connections, mainly on psychophysiological, physiological, and physical parameters, demonstrating that this inspiratory load cannot be considered placebo as pointed by literature^8,9,14,18^, at least to high-performance judo athletes studied here. Thus, this scenario can be applied as a prior task in the athlete's organism preparation for combat.

Interesting results were observed in the IM_PA_40 scenario. In this case, both centrality metrics (Degree and Pagerank) indicated technical-tactical parameters in the top-five nodes ranking. For example, the combat frequency (CF) and average combat time (ACT) so important to judo performance, occupied the 2nd position in the classification of Pagerank together with the time to reach the peak of blood lactate (TRLac). Considering that this metric represents the influence of one node on the others in the network, the highlighted technical-tactical nodes confirm the positive effect of the IM_PA_40 on judo match. These findings obtained through an integrative analysis detail the effects of IM_PA_, reinforcing the multifactorial aspects that can determine competitive success in judo^54^ and suggest acute strategies to assist coaches and athletes in training and competition.

Despite our study providing promising and positive effects of the IM_PA_ to judo matches, some limitations must be considered. First, we investigated only ten judo high-performance athletes. In next opportunities, we suggest increasing the sample size, as well as extending this protocol to different levels of competitive athletes. In addition, after this detailed investigation into the application of IM_PA_ in a single judo match, we point out the suggest to expand this investigation to assess the effects of IM_PA_ on successive matches as it occurs in a competitive way.

In summary, our study suggests the use of IM_PA_40 as a safe, legal, and non-invasive resource that plays a positive role in the judo match. Based on the integrative analysis by complex network model, IM_PA_40 increased connectivity and the influence of physical and technical-tactical parameters, and highlighted the important combat nodes to support performance in judo. According to the centrality metrics, IM_PA_15 also stimulates interactions among psychophysiological, physical, and physiological parameters. These results confirm the positive effect of the IM_PA_ in the judo modality, pointing out this strategy to prepare the organism (IM_PA_15) and to improve performance (IM_PA_40) in judo match.
